# ‘Ritualistic’ examiner training and calibration

**DOI:** 10.1111/medu.70345

**Published:** 2026-10-19

**Authors:** Walter Tavares

**Affiliations:** ^1^ Department of Health and Society University of Toronto Scarborough Toronto Ontario Canada; ^2^ The Wilson Centre, Temerty Faculty of Medicine University of Toronto Toronto Ontario Canada

## Abstract

Examiner training can become “ritualistic” when consistency is pursued without clarifying what assessor preparation is meant to achieve. Perhaps a new ritual is needed. #MedEd

Assessment of clinical competence is a central aspect of health professions education and sufficiently complex that it warrants human judgement. Even as methods evolve, assessing performance in simulation and in practice continues to require observers to effectively notice, interpret, and judge behaviour in context and to translate it into something meaningful. This makes the preparation of assessors important, and given this reliance on human judgement, the field continues to develop strategies to support assessors' contributions.^1^, ^2^ The scoping review by Thampy et al. (2026), which aims to map and synthesise simulation‐based rater training and calibration efforts, is therefore timely.^3^

The scoping review is valuable less for what it settles than for what it exposes. Its central finding is not simply that examiner training produces mixed effects, but that the field continues to pursue a familiar intervention despite limited clarity about its effectiveness and what problem the intervention is meant to solve. In that sense, the review is less a verdict on examiner training than a diagnosis of a deeper conceptual uncertainty. It shows that examiner calibration is often treated as an unproblematic process, even though the empirical effects are inconsistent, the mechanisms are under‐specified, and the intended outcomes vary among score convergence, improved judgment and more defensible decision‐making. Revealing that uncertainty is meaningful, if not sufficiently attended to, then ‘standardisation’ becomes the default, even when it is not the most defensible or worthy goal.

This is the point at which the review becomes even more conceptually interesting. Much of the examiner training literature reviewed implicitly assumes a post‐positivist or psychometric logic: variability is error, consistency is quality and calibration is justified insofar as it reduces dispersion. That logic is coherent, but only within a particular view of assessment. As argued in the ‘compatibility principle’^4^ and elsewhere,^5^, ^6^ assessment features are not neutral technical components; they are commitments about what competence is, how it can be known and what kinds of claims can be justified from the resulting data. When those commitments are not aligned, assessment programmes may be conceptually incoherent even if their methods are well executed. Examiner training is no exception. If a programme assumes that rater judgement is an approximation of an underlying truth, then training aimed at convergence may make sense. However, as the review demonstrates, we continue to struggle with this effort, and assessment science has broadened to question this approach as a solution. If the assessment programme treats examiner judgement as interpretive, context‐sensitive and partly constitutive of the assessment act itself, then the goal cannot simply be to make raters think alike, especially because it does not seem to be possible or sustainable. Our health professions education community has recognised that examiner judgement is often not a simple measurement operation, raising questions about the utility of rater training and calibration efforts in our contexts.

The persistent finding that rater training has strong limitations is made even more problematic when we consider what has been learned about how raters engage in and behave during these cognitive activities. For instance, previous research on rater cognition suggests that observation, processing and integration are distinct but interdependent phases of the judgement process, and that each is shaped by selective attention, schema use, context and prior experience.^7^ Raters do not passively record performance and then report it. They engage in socially constructed interpretations.^8^ They actively simplify, infer, compare, weight and synthesise variably.^9^ They attend to different aspects of the same performance, derive different meanings from similar behaviours and translate those meanings into scores and narrative judgements through cognitive strategies that are both constrained and idiosyncratic.^10^, ^11^ This research carries at least two consequences. First, treating rater variability as noise to be removed is itself a philosophical stance, and one that is difficult to sustain in many contexts. Second, engaging humans as assessors may mean designing around how human judgement in assessment actually happens.

That may explain why efforts to train away variability often disappoint. The issue is not that assessors are unskilled or unwilling to comply, or that frame of reference or other forms of training are just poorly designed and implemented. It is that assessment is an idiosyncratic and cognitively difficult task navigated in a social context; many constructs in health professions when translated into assessment activities may ask too much of observers or include the wrong expectations.^12^, ^13^ The lesson is that assessment design, and the preparation of assessors, must respect what raters can realistically do, given the construct, task structure and context, and how they naturally engage.^14^

Seen in that light, the authors' conclusion that there is widespread endorsement of examiner training and calibration, but a weak empirical foundation, is not surprising. Identical scoring is not the same as shared reasoning; reduced variability is not the same as improved discernment; and a more defensible assessment system is not necessarily one in which all examiners behave the same way. The review implicitly points to these tensions, but the field still tends to collapse examiner training and calibration processes and outcomes into single metrics of reliability or accuracy or to enforce convergence broadly. That collapse is theoretically too crude and, in many instances, not compatible with assessment in our context.

This does not mean assessor preparation should be abandoned. On the contrary, the review reinforces the need for it. But the object of preparation needs to change. Rather than trying to teach examiners to score identically, we should help them understand the assessment task, recognise the kinds of evidence that matter in a given context, explore the presence and impact of social biases (not to be confused with error) and become aware of the interpretive moves they habitually make and why.^15^ That includes being explicit about the intended use of the data, the nature of the construct and the role of examiner judgement in the overall assessment design. It also means extending readiness beyond psychometric outputs and examining how assessor preparation promotes coherence of reasoning, educational or validity value, fairness, equity, transparency, quality of the contribution, or construct representation.

A more defensible response is to reframe efforts as assessor readiness rather than rater training or calibration.^15^ The distinction is not semantic. As Thampy et al. (2026) point out, training and calibration imply that assessors should be modified to conform to a desired standard of judgement. Readiness implies something different: that assessors should be prepared to contribute meaningfully to an assessment system whose epistemic logic has already been made explicit and aligned.^4^ Readiness is about assessment designers understanding their context, assessors and learners, and helping assessors understand the role they are meant to play, the kinds of information they are expected to use, the limits of their own cognition and the downstream uses of the data they generate. It is not about forcing agreement for its own sake or to make claims about accuracy. It is about enabling assessors to work productively within the intentions of the assessment programme while acknowledging and working with human cognition and contextual influences.

Several implications come from the scoping review on examiner training and calibration. It should not be read as evidence that examiner training has failed and therefore requires remediation. Instead, it signals that the field has not yet settled the philosophical question of what examiner preparation is for, or how we continue to limit the use of research on rater cognition, or perhaps that our community has clung too tightly to a comfortably familiar approach. As such, standardisation, attempts to promote accuracy, and efforts to shape thinking appear to remain the default solution, even where they are conceptually and empirically misaligned with assessment science in health professions education. As Thampy et al. (2026) put it, rater training and calibration can become ‘ritualistic’: something carried out because the field expects it, not because its rationale has been sufficiently articulated or that there is sufficient evidence to support it. Their scoping review provides enough evidence to make that concern plausible. The more defensible path is not to routinely attempt to make raters think alike but to promote their readiness in a philosophically aligned and compatible assessment system designed to responsibly use what different assessors notice, infer and value. That should become the ritual.

## AUTHOR CONTRIBUTIONS

WT conceived the argument, reviewed the relevant literature, drafted the manuscript and approved the final version.

## CONFLICT OF INTEREST STATEMENT

The author has published previous scholarship on assessor cognition and rater training that is cited in this commentary. The author declares no financial conflicts of interest.

## Data Availability

Data sharing not applicable to this article as no datasets were generated or analysed during the current study.
